# Tridimensional Changes in Mandibular Arch after Rapid Maxillary Expansion Therapy: A Clinical Study

**DOI:** 10.3390/children10050775

**Published:** 2023-04-25

**Authors:** Giuseppina Laganà, Valeria Paoloni, Chiara Pavoni, Daniel Palmacci, Arianna Malara

**Affiliations:** 1 Department of Systems Medicine, University of Rome ‘Tor Vergata’, Viale Oxford 81, 00133 Rome, Italy; 2 UniCamillus—Saint Camillus International University of Health Sciences, Via S. Alessandro, 8, 00131 Rome, Italy

**Keywords:** digital models, arch form, expansion, RME, tooth movement

## Abstract

**Aim**: The upper jaw transverse deficit is certainly one of the most common clinical issues in the orthodontic field. It can be due to skeletal or dental factors, and its etiology may be both genetic and environmental. Rapid maxillary expanders (RMEs) are certainly the most effective appliance for upper transverse deficiency correction. The aim of this study was to evaluate the changes that occurred in the mandibular arch during treatment with RMEs in growing subjects by analyzing tridimensional lower digital casts. **Materials and Methods:** The study group (SG) consisted of 20 subjects (10 M, 10 F; mean age 9.4 ± 2.8 years old) randomly selected at the Department of Orthodontics at the University of Rome, “Tor Vergata”. The inclusion criteria were negative posterior transverse interarch discrepancy ≥ 4 mm, mixed dentition phase with first permanent molars erupted, and prepubertal skeletal maturation stage (CS1-2), evaluated on a lateral radiograph through the Cervical Vertebral Maturation method. The SG was compared to an untreated control group (CG) of 20 subjects (10 M, 10 F, mean age 8.7 ± 2.3 years old) enrolled with the same inclusion criteria. The SG was treated by using RMEs. Dental casts of the lower arch were taken at two different times (T0–T1 = 6 months). All the dental casts were scanned with an OrthoXscan (Dentaurum 6mmbh E Co., Ispringen, Germany) and twenty points on the mandibular arch were digitized using Viewbox software. A Student *t*-test was used to compare the means of the quantitative variables associated with the effect of the device over time T0 and T1. **Results:** The results show a statistically significant increase (*p* < 0.05) in the intercanine and the intermolar diameters between the times T0 and T1 when compared to the CG. **Conclusions:** Rapid maxillary orthopedic expansion may achieve an increase in mandibular intercanine and intermolar diameter.

## 1. Introduction

The upper jaw transverse deficit is certainly one of the most common clinical issues in the orthodontic field. It can be due to skeletal or dental factors, and its etiology may be both genetic and environmental [1]. Upper jaw constriction could be related to various disorders, such as occlusal and aesthetic disharmony, but also functional difficulties including the narrowing of the pharyngeal airway, increased nasal resistance and alterations in tongue posture, leading to retroglottic airway narrowing and mouth breathing [2].

Narrow maxillary arches and posterior crossbites are recurring malocclusions in the primary and mixed dentition, with an incidence between 7 and 23% [3]. They often appear unilaterally as a result of a functional shift of the mandible to the side of the crossbite [4].

The treatment of maxillary dento-skeletal constriction consists of opening the mid-palatal suture and of expanding the roof of the mouth and the floor of the nose [5].

Skeletal expansion can correct a posterior crossbite, if present, gain space in the jaw, and the underlining permanent tooth buds can be buccally repositioned [6].

The rapid maxillary expander (RME) is certainly the most used appliance to correct upper transverse deficiency. The device was introduced at the end of the 19th century, but only became popular in the late 1960s, thanks to the investigations of Dr. Haas. Several modifications to the original expander design have been proposed since then. Among these, the replacement of the original maxillary palate expander with fixed, tissue-supported acrylic splints, and hygienic all-metal frame expanders (Hyrax) have become very popular in orthodontics [7]. The safety and reliability of the palatal expansion have been persistently reported in the orthodontic literature. The primary objective of the treatment with RMEs is to provide the opening of the mid-palatal suture, giving an adequate and steady maxillary width increase through the application of heavy and intermittent forces in a short period of time [8].

RME was not only employed to increase the maxillary transversal dimension, but also to create supplementary space in the narrowed maxillary dental arches, in order to solve upper crowding [9]. In addition, after the opening of the mid-palatal suture, not only transversal, but also vertical and anteroposterior changes were observed. One of the most prevalent effects was the clockwise rotation of the mandibular plane, a consequence of the inferior–posterior position of the mandible after the application of RMEs [8]. Despite the available literature regarding maxillary expansion and its effects on the maxillary bone and on mandibular repositioning, there are very few studies analyzing the mandibular dentoalveolar changes after maxillary expansion. Since the 1970s, spontaneous changes in the mandibular dentition have been reported after a maxillary expansion, despite the high resistance of the mandibular bone [10]. Haas asserted that a permanent increase in the maxillary apical base causes a spontaneous, permanent, and important increase in mandibular arch width [11,12].

Several years later, Grassia et al. performed a comparison between rapid and mixed maxillary expansions by evaluating arch changes on dental casts. In both groups, significant increments of the upper and the lower jaws, in terms of arch widths, were found at the end of the treatment [12].

However, the effects of the maxillary expansion on the mandibular arch are still controversial, considering that most of the studies evaluated the dental and the skeletal effects of RMEs using bidimensional analysis on lateral and posteroanterior cephalometric radiography or on dental casts [13].

Therefore, the objective of the current study was to evaluate the changes that occurred in the mandibular arch during the treatment with rapid maxillary expanders (RMEs) in growing subjects by analyzing tridimensional lower digital casts, and to compare these results to an untreated control group. The hypothesis of the current investigation is that there are spontaneous changes in the mandibular arch after RME therapy.

## 2. Materials and Methods

This study was carried out in accordance with the principles set out by the World Medical Assembly in the 2008 Declaration of Helsinki on medical protocols and ethics, and it was approved by the Ethics Committee of the University of Rome “Tor Vergata,” (protocol number: 140/19). Written consent was obtained from all the parents of the subjects included in this study.

The study group (SG) of this retrospective investigation consisted of 20 subjects (10 males, 10 females; mean age 9.4 ± 2.8 years old) randomly selected from the Department of Orthodontics at the University of Rome “Tor Vergata,” from January 2021 to December 2021. The inclusion criteria were as follows: negative posterior transverse interarch discrepancy ≥ 4 mm [14], mixed dentition phase with first permanent molars erupted, and prepubertal skeletal maturation stage (CS1-2), evaluated on a lateral radiograph through the Cervical Vertebral Maturation (CVM) method [15]. Patients with previous orthodontic treatments, dental anomalies, permanent dentition, syndromes, or systemic pathologies were excluded from this study.

The SG was compared to a control group (CG) of 20 subjects (10 males, 10 females, mean age 8.7 ± 2.3 years old) enrolled with the same inclusion criteria and who did not undergo any orthodontic treatment for the duration of this study. The non-treatment of the CG was due to caries problems at the dental elements on which the orthodontic device was anchored. Such patients were sent for dental treatment before starting the treatment.

### 2.1. Treatment Protocol

**Figure 1:**
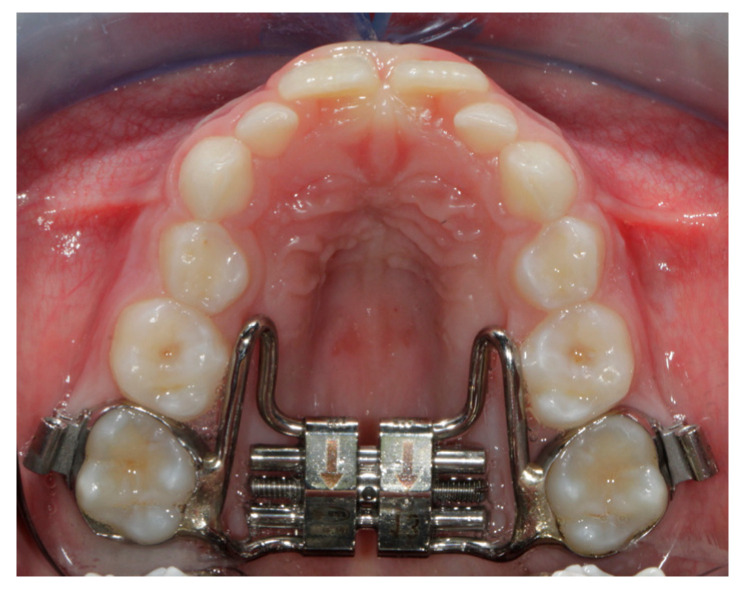
Butterfly Rapid Maxillary Expander.

All the subjects of the study group (SG) underwent treatment with the Hyrax butterfly Rapid Maxillary Expander (RME) with Leone^®^ screw with bands cemented on the upper permanent first molars (Figure 1).

The reliability of palatal expansion has been persistently reported in orthodontic literature [7,16]. However, a high incidence of external root resorption has been observed in abutment teeth after RME therapy [17]. To improve the orthopedic effect, the expanders, from the beginning, were equipped with bands in the maxillary first premolars and in the first molars [18]. In order to simplify the technique, the bands in the first premolars were substituted with an arch anchorage in several expander models [19]. Banded teeth had a different load than wire teeth, and this difference may have mechanical and biological effects that have not yet been fully investigated [16]. The selected patients had a single expansion phase performed on them, keeping the expander for six months. Once the device was fitted, the activation began, and it was completed whenever the overcorrection was reached with contact of the palatal cusps of the upper molars with on the buccal cusps of the lower molars [20].

The expansion protocol of the rapid butterfly expander consisted of two activations per day for the first week (0.4 mm) and one activation (0.2 mm) per day for the following week until upper molars hypercorrection was achieved. The lower arch did not undergo any orthodontic treatment.

Dental casts of the lower arch for both the SG and the CG were taken at two different times. For the SG:

- T0: before the treatment;
- T1: at the end of treatment, mean time six months after the last activation of RME.

For the CG:

- T0: at the first clinical check;
- T1: six months after the first visit.

All of the lower dental casts were scanned with an OrthoXscan (Dentaurum 6mmbh E Co, Ispringen, Germany) with a resolution < 20 microns. Twenty points on the mandibular arch were digitized using Viewbox software and the following measurements were analyzed:

- Mandibular intermolar width (L6–L6): distance between the tips of the distobuccal cusps of right and left mandibular first permanent molars;
- Mandibular intercanine width (L3–L3): distance between the tips of the cusps of right and left mandibular deciduous canines.

Lower (L6) first permanent molar buccolingual inclinations (BLIs) were measured. To assess the inclination of the teeth, an optimal occlusal plane was defined passing through the tips of the buccal cusps of the first permanent molars, first and second deciduous molars, decussate canines, and the incisal margins of the lateral and central incisors. This plane was utilized as a landmark to generate a further reference plane: the paracoronal plane, which was perpendicular to the occlusal plane. A curve passing through the long axis was drawn for each tooth analysed and the line of best fit was set using the most occlusal and gingival points of the curve as a reference. The inclination of the tooth was obtained from the angle formed between the line of best fit of each tooth and the paracoronal plane [21]. The inclinations of the lower right first molar and the lower left first molar were measured.

### 2.2. Statistical Analysis

To determine the reproducibility of this method, 20 digital casts were re-digitalized by the same operator (dr. AM) 10 days after the first digitization.

To confront the two measurements (systematic error), a paired *t*-test was applied. The amount of random error was determined according to the moment estimator method [22]. The strength of this study for the independent-sample *t*-test was assessed on the basis of the sample size of the two groups and an effect size of 0.9 [23]. The power was 0.80, with an alpha level of 0.05 (SigmaStat 3.5, Systat Software, Point Richmond, CA, USA). Since the data were normally distributed, a paired *t*-test was chosen to compare T1−T0 variations. The significance level was set at 5%. The software chosen for data analysis was SPSS (Statistical Package for the Social Sciences), version 18.0 (IBM Corp, Chicago, IL, USA).

## 3. Results

Among the repeated digital measurements, no systematic error was observed. The precise definition of the selected points and the presence of a previously trained examiner allowed for the reduction of the systematic error. The average random error was 0.41 mm and remained within acceptable limits since the software was able to provide a more accurate view of the anatomic details.

The study group (SG) consisted of 20 subjects, of which 10 were males and 10 were females. The average age of the study group was 9.4 ± 2.8 years old. The control group (CG) was composed by 20 subjects, of which 10 were males and 10 were females, mean age 8.7 ± 2.3 years old (Table 1).

The data were calculated at the two different times (T0, T1) and were compared in both the study group and the control group. In the SG, the following results can be observed (Table 2): an increase in the average of intercanine diameter (*p* = 0.04) and intermolar diameter (*p* = 0.01), and a decrease in the torque of both first lower molars (3.6, 4.6). In the short term (T1 − T0 = 6 months). The analysis of the results showed statistically significant changes in all evaluated parameters (*p* < 0.05).

In the CG, no significant differences between the mean of the right-lower first molar torque and of the left-lower first molar torque were observed. Neither the increase of the average of the intercanine diameter or of the intermolar diameter were statistically significant (Table 3).

## 4. Discussion

A great deal of research has been conducted on rapid maxillary expansion (RME) over the last century. It is now widely accepted that this device causes an opening of the mid-palatal suture by using large forces and it produces detectable changes in the maxillo-facial structures [24].

The primary aim of this clinical retrospective study was to evaluate the changes that occurred in the mandibular arch during rapid maxillary expansion treatment in growing subjects, through the analysis of tridimensional lower digital casts, comparing these results to an untreated control group.

The quantitative results obtained from this investigation of the inter-arched changes varied by view. This may be due to different post-treatment phases, the distribution of the sample, the skeletal Class, or the type of malocclusion in the different studies [25]. The short- and long-term dento-skeletal effects of the rapid expansion of the upper jaw have already been analyzed in various reviews and meta-analyses [26,27,28,29,30,31]. For instance, in 2009, Ballanti et al. demonstrated through a CBCT study that the rapid expansion of the upper jaw produced a significant increase in the maxillary transverse dimensions at the crown and at the apex of the first molars [32]. The protocol involved two activations per day for 14 days, until 7mm of expansion was achieved. Thereafter, the expander remained in place as a passive retainer for at least six months. Another result of this paper was that, at the end of the active expansion phase, the buccal bone plate thickness of the supporting teeth showed a significant decrease; after the retention period, a recovery of both buccal and lingual plate thickness was observed.

Regarding the lower arch, previous studies have shown a slight but continuous decrease in the intercanine distance (0.5–1.5 mm) during the second dentition [33]. Contrarily, Moorrees and Reed showed that the intercanine diameter does not change for 8- to 10-year-olds, while the intermolar diameter increases by 3–4 mm for 6- to 17-year-olds [34]. Another relevant study showed a significant increase in the mandibular perimeter maintained even 6.1 years after the rapid maxillary expansion treatment [35]. The arch perimeter was assessed as the length of the curve from the mesial surface of the mandibular permanent first molars, bisecting the contact points of the deciduous molars or premolars and canines, and smoothly fitting on the incisal edges of the anterior teeth [35]. Grassia found that RME can be considered an effective treatment option to improve transverse arch dimensions and gain space in both dental arches [12]. In his investigation, it was proved that in patients who underwent upper jaw expansion treatment, a significant increase in the maxillary intermolar, inter second premolar, inter first premolar and intercanine widths were found. Moreover, there was an increase in the mandibular intermolar and inter second premolar widths. No significant changes in the upper and lower arch depths were observed at the end of the treatment.

O’Grady et al. proved that RME combined with a mandibular removable Schwarz plate induces a significantly favorable increase in the transverse width of the mandibular arch [36]. In his work, Ugolini et al. evaluated the spontaneous mandibular response to RME therapy fifteen months after expansion in prepubertal patients [37]. The research revealed an important increase of about 1.9 mm for the mandibular intermolar width. The subjects who belonged to the control group showed, instead, a tendency for the transverse dimensions to contract, and a decrease in the angulation values of the molars, canines and lower incisors. Santos found an increase of 0.054 mm in the lower intermolar distance following the expansion of the maxillary arch by using a modified Hyrax appliance with the 7 mm expanding screws, “Dentarum” [38].

Leonardi et al., in a CBCT study, demonstrated that youngsters with a posterior unilateral crossbite present a very slight mandibular asymmetry before treatment; following the upper jaw expansion using an RME device, there was a small increase in the hemi-mandibular volume on the crossbite side compared with the non-crossbite side, attenuating the very mild volumetric asymmetry [39]. To obtain the hemi-mandibular volumes, the 3D mandibular models were separated into two sides. A plane tangent to the arbitrary mandibular occlusal plane and going through the most distal point of the equator of the second molar (ESM), was used as a cutting plane to separate the mandibular 3D model and to obtain the hemi-volume measurements [39]. These data were calculated through measurements made before and after treatment. During the data collection, it is essential to consider both spontaneous and induced alveolar changes caused by the device [40]. In the present research, in fact, the sample selected was studied in two different moments, before and after the expansion treatment. This investigation selected all the subjects before the pubertal stage, as it is known that the expansion device induces greater and more significant skeletal effects both at the mandibular and maxillary level if used in the prepubertal phase [37]. Although the literature has provided indications regarding changes in the lower arch after rapid expansion of the upper jaw, only a few studies have been performed with visualization and three-dimensional measurements [35]. Three-dimensional imaging allows us to view the selected sample more carefully. Data archiving is also easier, and the method for data collection is faster. The measurements made after the digitalization of the 20 points on the 3D models were entirely made by the same virtual program (Viewbox 4) without any operator error. The choice of 20 points on the models also allowed an accurate and rigorous analysis. In the present study, after the rapid expansion of the upper jaw was performed using an RME, the intermolar and intercanine diameters of the lower arch increased significantly. A significant increase in the intercanine (+1.28 mm) and intermolar (+1.62 mm) diameters was obtained throughout the duration of the treatment (T0–T1) when compared to the CG. The results of this study, therefore, confirm what has already been reported in the literature: the rapid maxillary expander is a reliable orthodontic device not only for the correction of the upper jaw constriction, but also for improving transverse arch dimensions and for gaining space in both the upper and lower arches. In particular, as well as the already known increase of the intermolar diameter, a spontaneous increase of the inter-canine diameter in the lower arch has also been demonstrated. The stated hypothesis is so confirmed.

This assumption is based on the consideration that the expansion of the maxillary dental arch causes an alteration of the balance between the forces of the tongue and of the cheek on the teeth of the lower arch. Therefore, the prevalence of the tongue forces on the teeth of the lower jaw should increase the mandibular dental arch width [35]. It is important to take these findings into account when clinically assessing the need for dentoalveolar expansion of the lower arch.

This study presents some limitation. First, this investigation was performed in a short-term period (six months), and the short interval time might bias the results, emphasizing the immediate dentoalveolar changes in mandibular structures influenced by maxillary expansion. Long-term studies and further investigations are needed. Second, this study is limited by its small sample size, which was due to the very restrictive inclusion criteria. Future goals will certainly include an increase in the sample size and a longer observation time.

## 5. Conclusions

The rapid maxillary expander (RME) is an effective and an efficient orthopedic treatment that can be employed to solve structural and functional problems. This procedure has been shown to have several positive side effects. Regarding the lower arch, the rapid maxillary orthopedic expansion acts indirectly on the lower molars and canines as well as inducing its now well-known effects on the upper arch.

## Figures and Tables

**Table 1:** Descriptive analysis.

	Number	Gender		Age
SG	20	10 M	10 F	9.4 ± 2.8
CG	20	10 M	10 F	8.7 ± 2.3

**Table 2:** Statistical data and measurements of T0 − T1 variables in SG.

Variables	T0	T1	T1 − T0	*p* Value
L3–L3	26.17	27.45	1.28	0.04 *
L6–L6	43.66	45.28	1.62	0.01 *
4.6 torque	45.52	42.40	−3.12	0.01 *
3.6 torque	44.12	40.91	−3.21	0.01 *

* Significant interaction; ns—not significant.

**Table 3:** Statistical data and measurements of T0 − T1 variables in CG.

Variables	T0	T1	T1 − T0	*p* Value
L3–L3	25.59	26.17	0.58	ns
L6–L6	43.10	43.66	0.56	ns
4.6 torque	44.41	46.52	−2.13	ns
3.6 torque	37.24	39.36	−2.12	ns

Significant interaction; ns—not significant.

## Data Availability

The datasets used and/or analyzed during the current study are available from the corresponding author on reasonable request.
